# Global Research Trends in EV-Based Cell-Free Therapy for Osteoarthritis: A Bibliometric Analysis

**DOI:** 10.1007/s13770-025-00749-2

**Published:** 2025-09-10

**Authors:** Chuanhui Zhang, Chen Xu, Chengshuai Sun, Weining Meng

**Affiliations:** 1 Department of Joint and Sports Medicine, Chaoyang Central Hospital, Chaoyang City, Liaoning Province China; 2 Department of Obstetrics and Gynecology, Chaoyang Central Hospital, Chaoyang City, Liaoning Province China

**Keywords:** Osteoarthritis, Cell-free therapy, Extracellular vesicles, Exosomes, Bibliometric analysis

## Abstract

**Background:**

Osteoarthritis (OA) represents a major global health challenge with no ideal treatment options available. Early-stage treatment typically focuses on symptomatic relief of pain and stiffness; while late-stage patients can only opt for surgical interventions such as joint replacement to improve quality of life. Cell-free therapy based on extracellular vesicles (EVs) has offered a novel therapeutic approach for regulating bone metabolism and repairing cartilage, demonstrating emerging potential.

**Methods:**

Publications related to OA and Cell-free therapy based on EVs were retrieved from the Web of Science Core Collection database from 1991 to 2024. Our study employed bibliometric methods to analyze publication trends, leading contributing countries, institutions, authors, journals, and emerging trends and research directions.

**Results:**

The analysis has revealed a rapid growth in publications since 2019. China dominated both in terms of publication output and citation counts. The most productive institution is Shanghai Jiao Tong University. The most prolific publishing outlet journal was the *International Journal of Molecular Sciences*, while in terms of citation impact, *Biomaterials* ranked first. De Girolamo, Laura from Aix-Marseille Universite and Ragni, Enrico from IRCCS Istituto Ortopedico Galeazzi shared the top position in publication output, while Noel, Daniele from the Universite de Montpellier was the author receiving the most citations. Research primarily clustered around key themes including: (1) therapeutic mechanisms of cell-free treatment based on EVs in OA, (2) research advances in cell-free treatment based on EVs, (3) exosome engineering, and (4) a novel drug delivery system for EVs.

**Conclusion:**

This is the first bibliometric study on cell-free therapy based on EVs for OA, providing orthopedic and regenerative medicine experts with comprehensive perspectives on the field's current status and future development directions.

## Introduction

The global healthcare landscape in the twenty-first century faces unprecedented challenges from chronic degenerative conditions, among which osteoarthritis (OA) emerges as a particularly prominent concern [1, 2]. In 2020, OA affected 595 million people, representing 7.6% of the global population. Over three decades, the incidence has surged by 132.2%, with projections indicating a further 60–100% increase by 2050 [3–5]. The major risk factors include age, female gender, excessive weight, and previous joint injuries [6]. Marked by deteriorating cartilage, hardened subchondral bone, and bone spurs, OA stands as the primary cause of mobility restrictions in adults and skeletal deformities in older populations [5, 7, 8]. Despite numerous early-stage treatments—from medications and injections to bracing and lifestyle changes—therapeutic outcomes remain suboptimal [9]. Failed conservative management necessitates surgical interventions like arthroscopic repair or joint replacement, which involve significant trauma and prosthetic limitations [10, 11]. These procedures also carry complication risks and generate substantial direct healthcare costs and indirect economic losses through reduced workforce participation [12, 13].

Recent research, including both animal studies and clinical trials, has validates mesenchymal stem cells as an efficacious treatment for cartilage injuries and osteoarthritis [14, 15]. The clinical application of mesenchymal stem cells is limited by rigorous protocols for harvesting and transportation, coupled with potential risks of infection and neoplasia [16–18]. With advancing tissue engineering research, studies reveal that mesenchymal stem cells function through paracrine effects, with their secreted extracellular vesicles modulating the intra-articular microenvironment and regulating cellular migration and proliferation [19, 20]. Moreover, due to their cell-free nature, these vesicles possess advantages including non-immunogenicity, non-tumorigenicity, and ease of storage and transportation [21–24]. Membrane-bound nanoparticles known as extracellular vesicles are secreted by cells and found throughout biological fluids. These structures facilitate intercellular signaling by conveying bioactive cargo—including proteins, lipids, and various nucleic acids (mRNA, miRNA, DNA)—thereby orchestrating physiological and pathological processes [22, 25]. Based on diameter and biogenesis mechanisms, they are classified into the exosomes, microvesicles, and apoptotic bodies [26]. Among various EVs subtypes, exosomes represent the most extensively studied population and have been shown to regulate osteoarthritic cartilage extracellular matrix metabolism, inflammatory responses, and angiogenesis. Their bioactive cargo, including proteins and nucleic acids, influences OA progression through paracrine or endocrine mechanisms targeting specific cells [27–29]. Research also indicates that microvesicles, like exosomes, can regulate OA chondrocyte metabolism and exhibit anti-inflammatory and chondroprotective effects, counteracting IL-1β's influence. MVs exhibit more pronounced effects, possibly due to Annexin A1 involvement, offering novel therapeutic strategies for joint diseases [30–34]. In the context of OA, EVs-based approaches have manifested particular promise, offering potential solutions for both disease modification and tissue regeneration [30, 35]. The scientific community's growing interest in the applications of EVs for OA treatment has catalyzed an unprecedented surge in research activities [23]. This expansion spans multiple scientific domains, encompassing fundamental biological investigations, innovation in organizational engineering [21], mechanistic studies of therapeutic effects [36], and early-stage clinical applications [37, 38].

Bibliometric analysis provides a powerful methodological framework for understanding the evolution and current state of scientific fields. Through quantitative assessment of publication patterns, citation networks, and collaboration structures, this approach reveals valuable insights into research trends and the knowledge development [39]. While several bibliometric studies have examined various aspects of regenerative medicine and arthritis research, the specific intersection of cell-free therapy based on extracellular vesicles (EVs) and OA treatment remains unexplored through this analytical lens. To bridge this research gap, we perform an extensive bibliometric investigation of the academic literature at the interface of Cell-free therapy based for EVs in OA. Our objectives encompass: (1) identifying major contributing entities, including countries, institutions, and research groups; (2) analyzing collaborative networks and research clusters; (3) mapping emerging research frontiers; (4) examining the chronological development and current status of research activities; and (5) providing evidence-based insights for future research directions. This investigation employs advanced bibliometric tools to generate a comprehensive understanding of the field's intellectual structure.

## Methods

### Source materials and search protocol

We obtained the study's bibliometric materials through the Science Citation Index Expanded (SCI-E) platform, accessed via Web of Science Core Collection (WoSCC) on January 18, 2025 [40]. To avoid bias, two investigators independently performed the literature search using the following search query: TS = (Osteoarthritis OR Degenerative Joint Disease OR Osteoarthrosis) AND TS = (Extracellular Vesicles OR Exosomes OR Microvesicles OR Secretory Vesicles OR Transport Vesicles) AND TS = (Cell-free therapy OR therapy). The search covered articles published from database inception to the search date. The study scope encompassed two types of publications: original research and review papers. From the WoSCC database, we obtained the full documentation and reference information for qualifying papers in plain text form. Key information including title, publication year, authors, journal, keywords, abstract, affiliations, and references was extracted for subsequent analyses. Any discrepancies in the search results were resolved through discussion and consensus.

### Bibliometric analysis

The compilation of fundamental metrics—publication numbers, regional distribution, institutional data, journal details, and authorship information—was performed using Microsoft Excel 2021The journals' impact factors (IF) and disciplinary categories were extracted from the 2023 edition of Clarivate Analytics JCR rankings. The visualization and analysis of bibliometric data were accomplished through CiteSpace (version 6.4.R1) [3], VOSviewer (version 1.6.20) [4], and the R software “Bibliometric Package” (version 4.4.2). We also used Scimago Graphica to visualize the data.

**Fig. 1:**
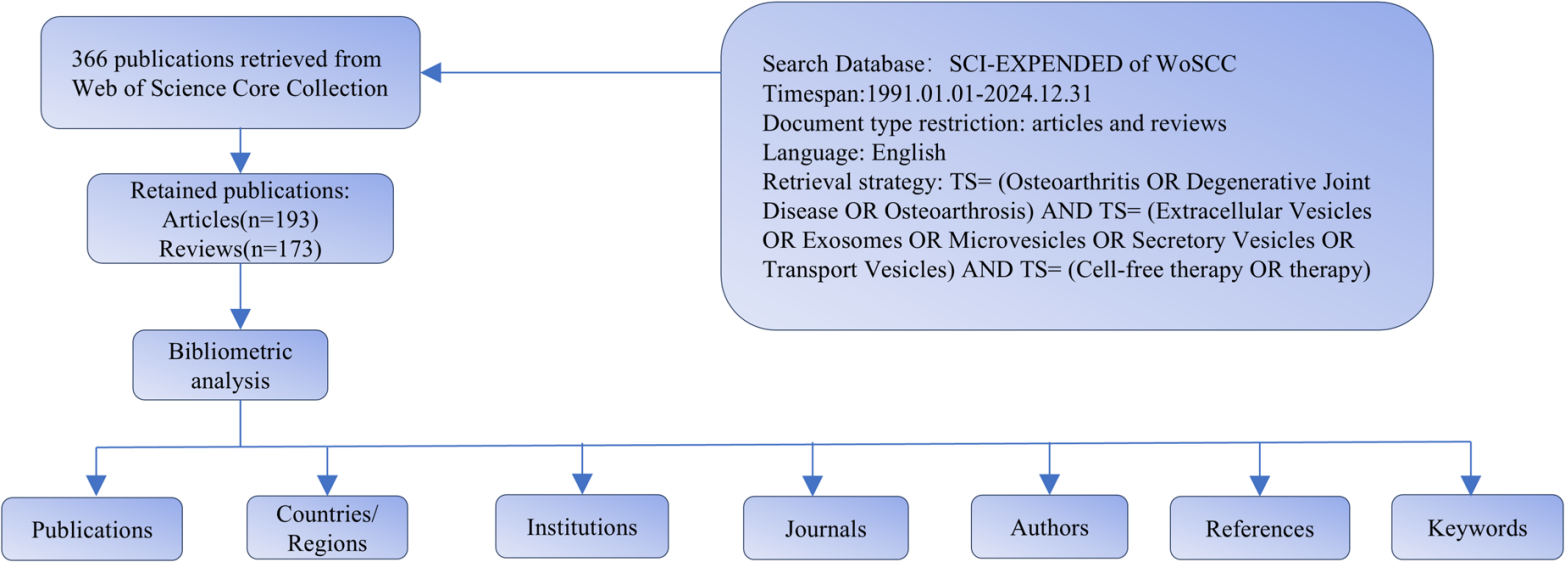
Flowchart of literature screening in this study

Built on Java architecture, CiteSpace serves as an analytical tool for exploring and displaying co-citation relationships. It can identify clusters, evolving trends, and landmark publications cross focused research areas via citation network evaluation and network visualization techniques. CiteSpace offers analytical capabilities for different bibliometric dimensions, including collaborative networks, keyword relationships, author citation patterns, and document reference associations [41, 42]. Similarly, VOSviewer functions as an analytical platform for generating and illustrating connections within bibliometric data [43]. It uses the VOS (Visualization of Similarities) mapping technique to translate complex bibliometric patterns into comprehensible visual displays. VOSviewer can create maps of authors, organizations, keywords, and terms based on co-occurrence, co-authorship, or co-citation data. In addition to these two main tools for network analysis and visualization, Bibliometrix, an R package, provides comprehensive bibliometric analysis and visualization functions [44]. It offers various functions for data importing, preprocessing, network creation and normalization, and statistical analysis. Bibliometric can generate numerous bibliometric indicators and plots, covering temporal publication data, influential entities (authors, journals, organizations), concept frequency metrics, thematic developments, and multi-variable relationship analyses. The three tools were selected due to their powerful features, ease of use, and wide application in bibliometric research. By combining their strengths, we aim to present an extensive and detailed examination of research contributions within OA and cell-free therapy based on EVs (Fig. 1).

## Results

### Analysis of publications

**Fig. 2:**
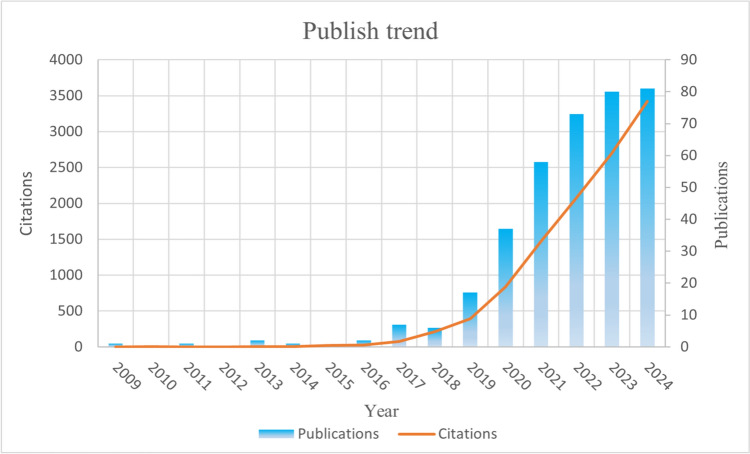
Annual publication volume and citation frequency of relevant articles

According to bibliometric data from Web of Science (WoS), which traces back to the first relevant publication in 1991, a total of 366 publications—consisting of 193 research articles and 173 reviews—have collectively garnered 5960 citations. The average citation frequency per article is 31.18, with 2222 authors publishing their research findings across 165 journals, utilizing 744 author keywords, and achieving an H-index of 54. The publication volume and citation patterns are illustrated in Fig. 2. Our analysis reveals that from 1991 to 2018, publications concerning OA and EVs were minimal. However, publication frequency exhibited exponential growth beginning in 2019, reaching its apex in 2024 (87 publications), followed by a stabilization period from 2022 to 2024. Overall, with the deepening understanding of OA and EVs, the significant role of cell-free therapy based on EVs in OA treatment has progressively garnered scholarly attention.

### Contribution by countries/regions

**Fig. 3:**
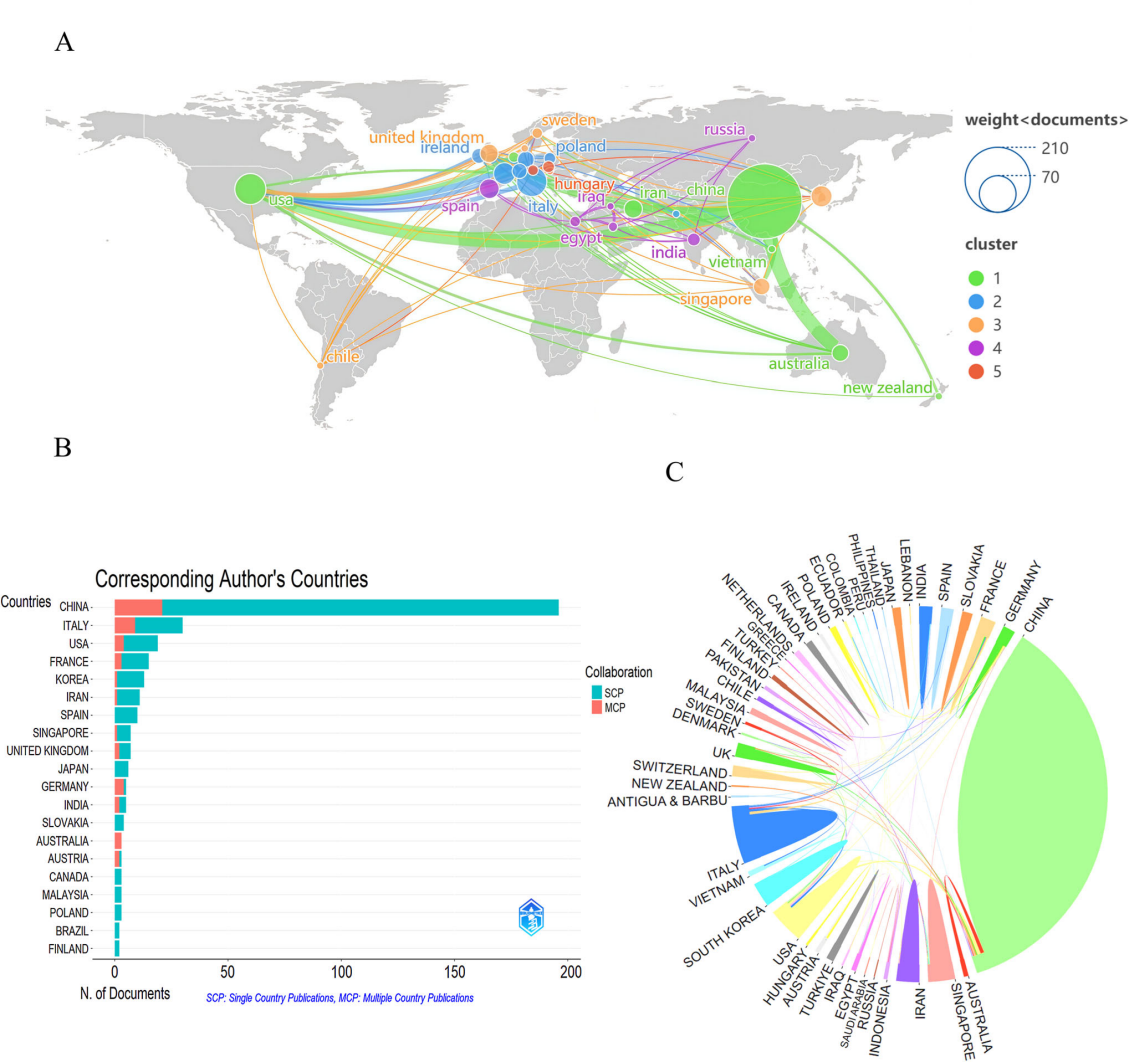
Analysis of countries/regions. **A** International collaboration network map. Node size represents publication volume, and countries with close collaborations are assigned the same color. **B** Distribution of corresponding authors by country. Same-Country Publication (SCP), indicating the number of papers published by authors from the same country, serving as an indicator of domestic research collaboration. Multi-Country Publication (MCP), indicating the number of papers published by authors from different countries, representing an important measure of international research collaboration. **C** Inter-country cooperation network. Sector area reflects research output, while line thickness indicates collaboration intensity

Investigations into cell-free therapy based on EVs for OA reflect contributions across 47 global countries/regions. Figure 3A illustrates the international collaboration patterns in research on osteoarthritis and cell-free therapies based on extracellular vesicles, revealing distinct clustering features and collaborative networks. China had the largest node, indicating the highest number of publications. The collaborative network is divided into five main clusters: Cluster 1 (green): centered around China and the United States, also including Asia–Pacific countries such as Singapore, Australia, and New Zealand. Cluster 2 (blue): characterized by regional collaboration among European countries, including Ireland, France, and Switzerland. Cluster 3 (orange): comprising countries such as Denmark and Germany. Cluster 4 (purple): represented by Spain. Cluster 5 (red): including Central European countries such as Austria. The thickness of the connecting lines shows that the most prominent network is the Asia–Pacific network centered around China and the United States (Cluster 1, green), which showed strong bilateral cooperation and extensive connections with Australia, New Zealand, and Southeast Asian countries. European countries form several smaller but interconnected networks, indicating a more fragmented research landscape with a tendency towards regional collaboration.

Figure 3B indicates the collaboration patterns of corresponding authors by country, categorized as Single Country Publications (SCP, shown in cyan) and Multiple Country Publications (MCP, shown in red). The data shows that although major research countries including China, the United States and Italy maintain strong publication records, most of them are the results of domestic cooperation, and cross-border research partnerships are insufficient. In contrast, although Germany has a relatively small number of publications, it exhibits a positive trend in international cooperation.

Figure 3C illustrates the research collaboration network among different countries. Arc lengths represent publication volume, while line thickness indicates collaboration intensity, with thicker lines denoting more frequent collaboration. China occupies a substantial arc length, indicating significant research output, while multiple connecting lines converge at both China and the United States, identifying them as principal hubs of research collaboration.

Table 1 presents the premier ten countries/regions ranked judging by the sum of publications and citation frequency. China led in publications (202 papers), succeeded by the United States (35 papers) and Italy (33 papers). Regarding citation frequency, China had a significantly higher citation count of 6,063 far surpassing other countries. Singapore follows with,557 citations, and the United States comes next with 1,052 citations. Additionally, high total link strengths are observed for the United States (41), China (33), and Germany (24), indicating that these countries have greater influence and importance.

**Table 1:** Top 10 countries/regions in articles, citations and total link strength

Rank	Countries/regions	Counts	Countries/regions	Citations	Countries/regions	total link strength
1	China	202	China	6063	USA	41
2	USA	35	Singapore	1557	China	33
3	Italy	33	USA	1052	Germany	24
4	France	17	France	1031	UK	22
5	South korea	16	Italy	651	Italy	20
6	Spain	14	South korea	495	Australia	17
7	Iran	12	Switzerland	367	Ireland	16
8	UK	12	Netherlands	343	Egypt	14
9	Australia	10	UK	284	France	13
10	Singapore	10	Australia	245	Switzerland	13

### Contribution by institutions

**Fig. 4:**
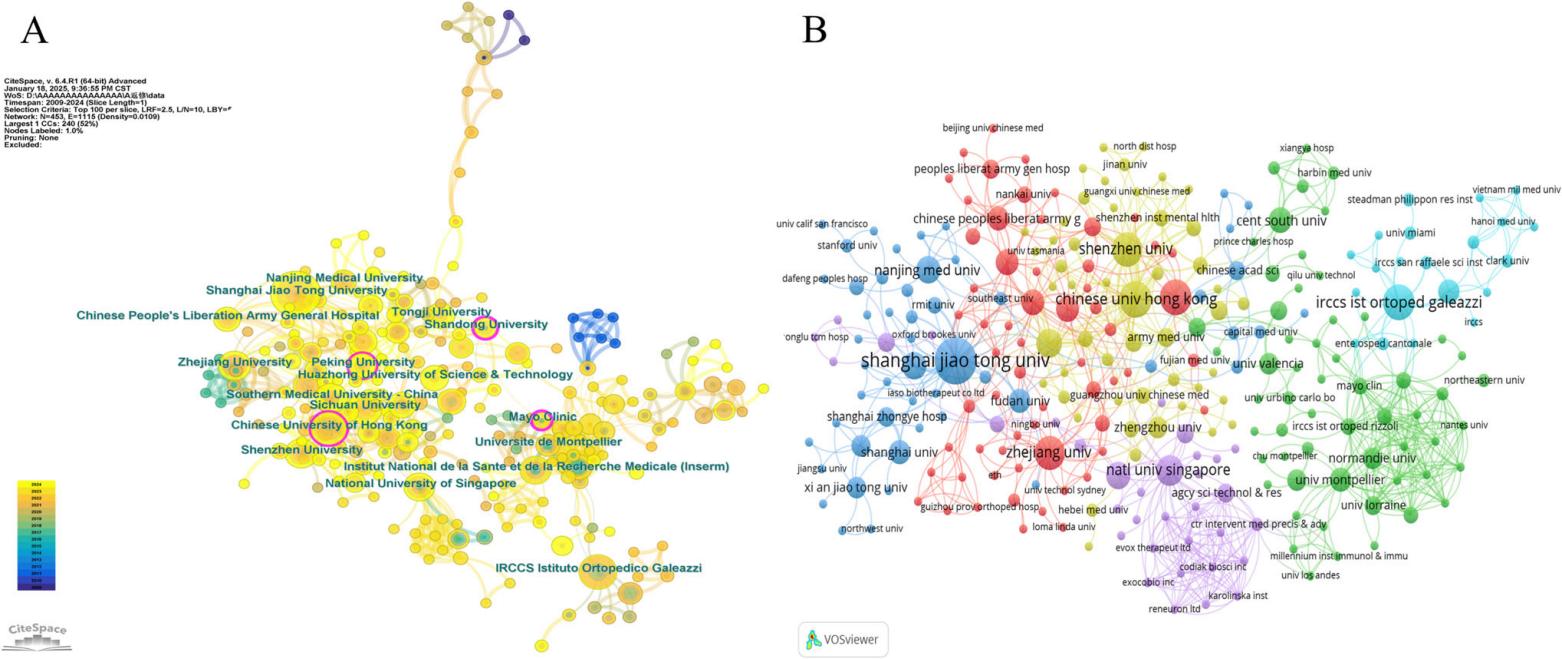
Analysis of institutional collaborations. **A** CiteSpace network map. Nodes represent institutions with size indicating research output; lines show collaboration strength between institutions; purple rings highlight nodes with high betweenness centrality (BC ≥ 0.1), indicating key network intermediaries. **B** VOSviewer co-authorship map. Node size reflects publication volume; connections indicate collaboration strength; institutions with close partnerships share the same color

A visualized diagram of institutional partnership networks was created using CiteSpace, as shown in Fig. 4A. The leading five institutions by publication volume were Shanghai Jiao Tong University (n = 25), IRCCS Istituto Ortopedico Galeazzi (n = 14), Chinese University of Hong Kong (n = 13), Sichuan University (n = 13), and Shenzhen University (n = 12). Four research institutions demonstrated betweenness centrality (BC) exceeding 0.1, with Shandong University exhibiting the highest centrality (0.26), chased by Mayo Clinic (0.25), Chinese University of Hong Kong (0.12), and Peking University (0.11). These institutions with high centrality serve as bridges, functioning as hubs for information flow and resource sharing.

Additionally, Fig. 4B presents the co-authorship analysis of institutions conducted using VOSviewer. Interestingly, two relatively independent clusters can be observed in the figure. The green cluster, centered around IRCCS Galeazzi Orthopedic Institute, has formed a tight European research network with IRCCS Rizzoli Orthopedic Institute, Université de Montpellier, and Université de Lorraine, demonstrating strong clinical research capabilities in orthopedics. The collaboration pattern of this cluster indicates a well-established clinical research framework. In contrast, the purple cluster, centered around the National University of Singapore, exhibits a unique academia-industry collaboration model, particularly through its connections with biotechnology companies such as Exocobio Inc and Zodiak Biosci Inc. The structure of this cluster reflects an emphasis on translational research and commercialization potential in extracellular vesicle therapy.

### Contribution by journals

**Fig. 5:**
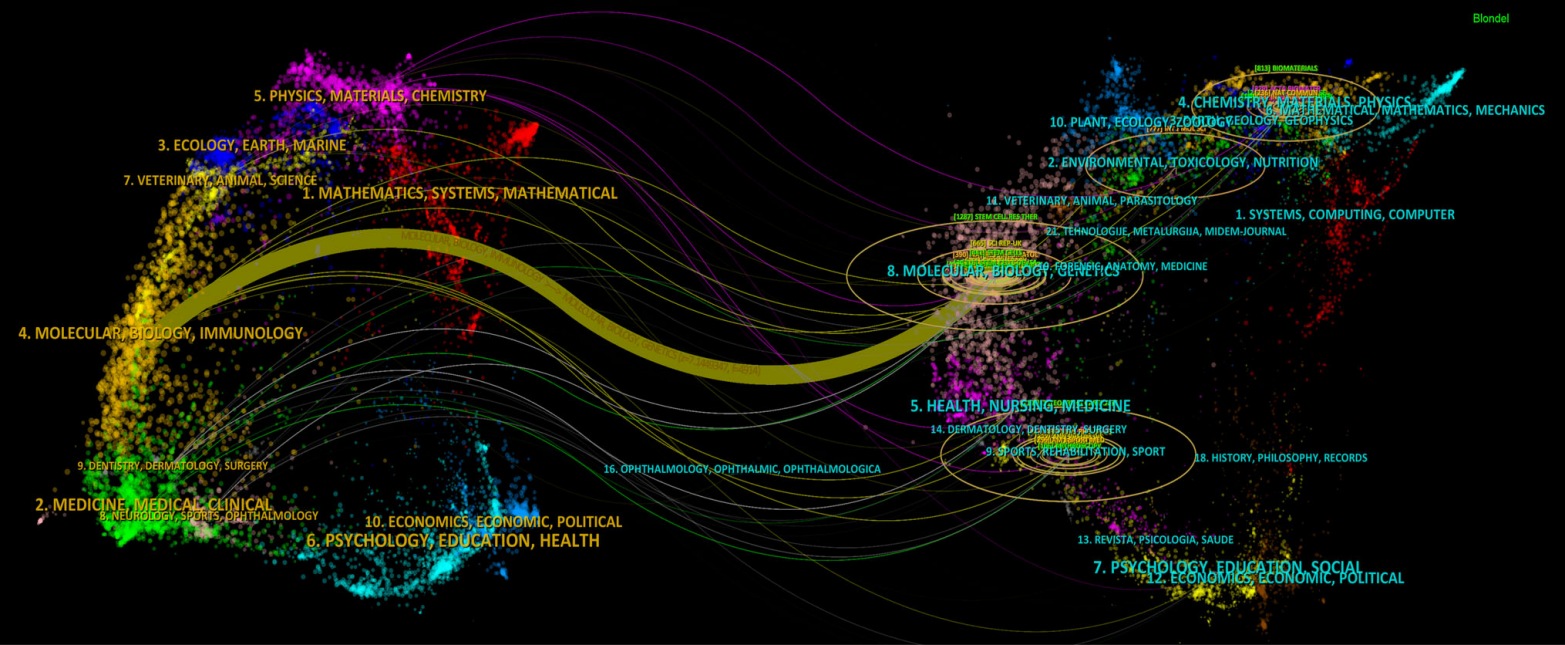
Analysis of academic journal citation networks using dual-map overlay. The left map shows citing journals, the right shows cited journals. Colored trajectories trace citation flows between research fields, with trajectory width indicating citation intensity—thicker paths represent stronger citation relationships

Visual analysis of published journals and co-cited journals was conducted to identify active and influential journals in the field of OA and cell-free therapy based on EVs. Across the academic landscape, 165 journals collectively published 366 studies relevant to this topic. Bradford's Law demonstrates that literature within a specific discipline concentrates in a limited number of core journals, while related publications disperse across numerous peripheral journals. This principle categorizes journals into three zones: core (few high-productivity journals), related (moderate number with moderate output), and peripheral (many journals with minimal contributions), following an approximate distribution ratio of 1:n:n^2^, where n is the Bradford multiplier. Applying Bradford's Law to our dataset, we identified the core journals in this research domain: International Journal of Molecular Sciences, Frontiers in Bioengineering and Biotechnology, Stem Cell Research & Therapy, Frontiers in Cell and Developmental Biology, Journal of Nanobiotechnology, Cells, Bioactive Materials, Journal of Orthopaedic Translation, Biomedicines, Stem Cells International, and Pharmaceutics. This identification serves as a valuable reference for researchers conducting literature reviews, research assessments, and bibliometric analyses, enabling more efficient targeting of primary information sources within the field. From Table 2, the following conclusions can be drawn: The distribution of papers displayed that *International Journal of Molecular Sciences* led with 26 contributions, with *Frontiers in Bioengineering and Biotechnology* publishing 19 works. This was trailed by *Stem Cell Research & Therapy* (12 papers), *Frontiers in Cell and Developmental Biology* (11 papers), and *Journal of Nanobiotechnology* (11 papers). From the premier ten co-cited academic journals, three received over 500 citations. *Biomaterials* (1057 citations) demonstrated the highest citation frequencies. Figure 5 presents a dual-map overlay of journals, demonstrating the publication patterns among individual scholarly venues, citation trajectory development, and research focus evolution. Overall, Fig. 5 exhibited citing journals on the left side and cited journals on the right side, using connecting pathways to indicate information transfer. The figure revealed a notable trend of interdisciplinary integration, forming a complex network of interdisciplinary connections with keywords interwoven from multiple disciplines including physics, chemistry, biology, mathematics, medicine, biomaterials, and economics.

**Table 2:** The number of publications, IF, and JCR quartile of the top 10 journals and cited-journals

Rank	Journal	Counts	IF (2023)	JCR	Cited-Journal	Citations	IF (2023)	JCR
1	International Journal of Molecular Sciences	26	4.9	Q1	Biomaterials	1,057	3.9	Q2
2	Frontiers in Bioengineering and Biotechnology	19	4.3	Q1	Stem Cell Research & Therapy	916	7.1	Q1
3	Stem Cell Research & Therapy	12	7.1	Q1	Frontiers in Bioengineering and Biotechnology	562	4.3	Q1
4	Frontiers in Cell and Developmental Biology	11	4.6	Q1	Osteoarthritis and Cartilage	492	7.2	Q1
5	Journal of Nanobiotechnology	11	10.6	Q1	Scientific Reports	458	3.8	Q1
6	Cells	9	5.1	Q2	Theranostics	451	12.4	Q1
7	Bioactive Materials	8	18.0	Q1	International Journal of Molecular Sciences	364	4.9	Q1
8	Journal of Orthopaedic Translation	8	5.9	Q1	Seminars in Cell & Developmental Biology	354	6.2	Q1
9	Biomedicines	7	3.9	Q2	Journal of Nanobiotechnology	350	10.6	Q1
10	Stem Cells International	7	3.8	Q2	Bioactive Materials	346	18.0	Q1

### Contribution by authors and co-cited authors

**Fig. 6:**
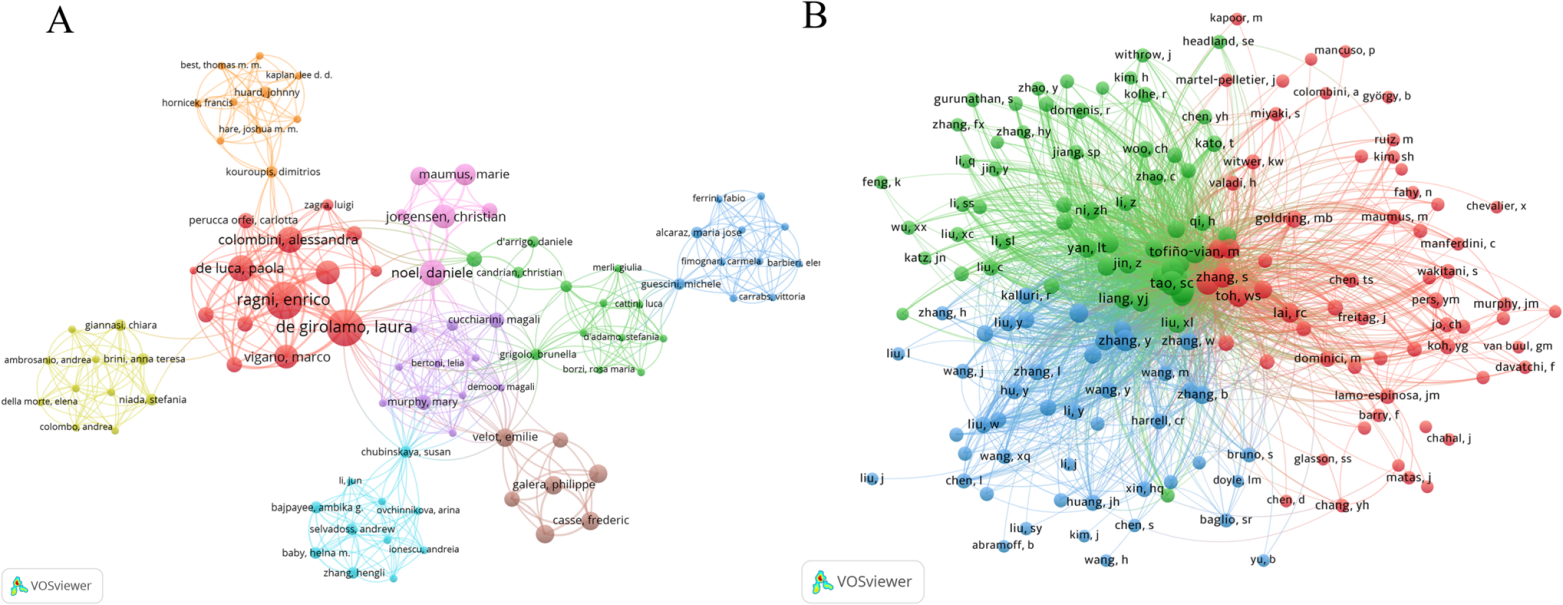
Analysis of author relationships. **A** Co-authorship cluster map. Nodes represent authors with size indicating publication count; line thickness shows collaboration intensity; color-coded clusters represent closely collaborating research groups. **B** Co-citation network. Node size reflects citation frequency or academic influence; line thickness indicates co-citation strength; same-colored authors are frequently cited together, suggesting similar research domains or complementary contributions

A cluster density graphical analysis of author co-authorship analysis was rendered using VOSviewer (Fig. 6A). Among the 2222 authors, De Girolamo, Laura from Aix-Marseille Universite and Ragni, Enrico from IRCCS Istituto Ortopedico Galeazzi shared the top position in publication output (12 papers). Furthermore, authors with close collaborative relationships were assigned to groups of the same color, forming a total of 9 author clusters. Among these, clusters comprising Chinese authors were the most numerous. Regarding co-citation analysis, 167 authors who were co-cited at least 20 times were included (Fig. 6B). The first three authors by total link strength (TLS) were Tao, SC (25,489), Zhang, SP (23,424), and Cosenza, S (21,550), indicate closer and more frequent contact with other researchers.

### Analysis of keywords

Keywords are typically the most representative terms selected to explain research topics and serve as key indicators in bibliometrics for assessing research concentration and shifts in research focus. Analyzing keyword variations reveals the primary research directions and evolutionary patterns of research hotspots within a field. We extracted 744 keywords from 366 articles and used VOSviewer software to build a co-occurrence network, identifying 94 keywords that appeared at least three times. The keywords were automatically categorized into four main functional groups. Cluster 1 centers on the pathological mechanism research of osteoarthritis, encompassing related pathological processes such as cartilage damage, rheumatoid arthritis, and osteoporosis, reflecting the fundamental research foundation in this field; Cluster 2 focuses on extracellular vesicles and links to emerging therapeutic strategies including drug delivery, gene therapy, and cell-free therapy, demonstrating innovative developments in treatment approaches; Cluster 3 primarily revolves around mesenchymal stem cells and tissue engineering, closely connected with biomarker and senescence research, highlighting the significant role of stem cell therapy in the field of osteoarthritis; Cluster 4 concentrates on the clinical translation of regenerative medicine, including application research in biomaterials, platelet-rich plasma, and adipose-derived stem cells. Notably, extracellular vesicles, as an emerging therapeutic carrier, have formed close connections with all three other clusters. This multidirectional cross-correlation suggests the vast application potential of cell-free therapeutic strategies based on extracellular vesicles in future osteoarthritis treatment.

**Fig. 7:**
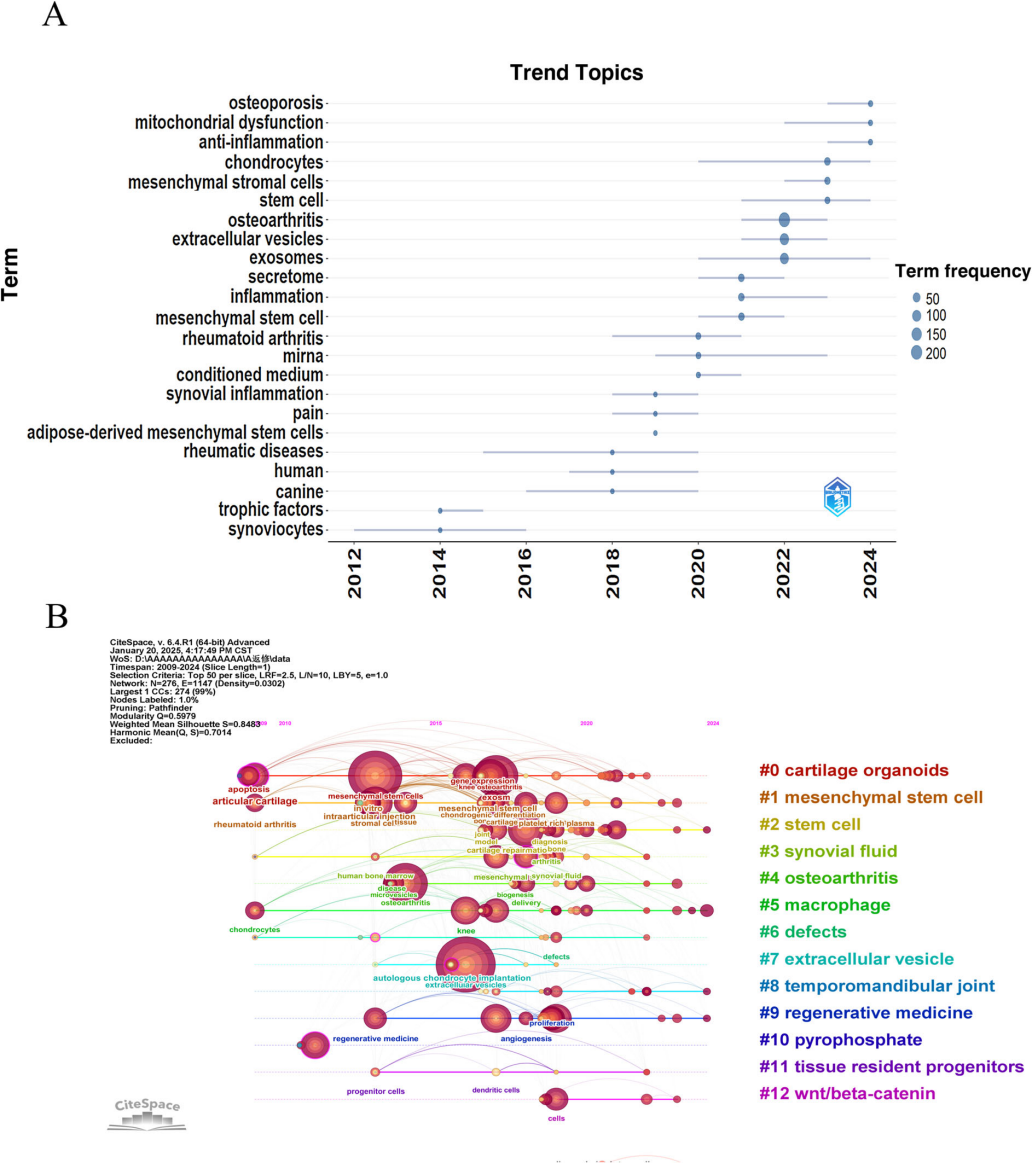
Keyword analysis. **A** Trending topics. This visualization shows the temporal evolution of research themes based on keyword frequency changes over time. **B** Timeline visualization of research trends. Keywords within the same cluster are arranged horizontally along the timeline, with earlier-appearing nodes representing foundational research areas, larger nodes indicating greater attention, and longer spans showing sustained research duration, thereby revealing the emergence, growth, and decline of research topics over time

Figure 7A, a keyword trend map, clearly illustrates the hot topics and dynamic changes in OA and cell-free therapy based on EVs. Notably, early research primarily focused on fundamental studies of synoviocytes and trophic factors, whereas in recent years, extracellular vesicles and exosomes have received considerable attention, along with increasing focus on mechanistic studies involving mitochondrial dysfunction and anti-inflammatory processes. This transition indicates a paradigm shift from traditional cell-based approaches to cell-free therapeutic strategies. Additionally, the increasing frequency of terms related to mesenchymal stromal cells and chondrocytes, coupled with attention to secretome and inflammation, reflects a deeper understanding of underlying disease mechanisms and potential therapeutic targets.

Figure 7B, constructed using CiteSpace software, illustrates the evolution trajectory and research hotspot distribution in this field over the past 15 years (2009–2024). The research themes demonstrate distinct temporal progression characteristics: early research primarily focused on articular cartilage pathology and basic research of apoptosis mechanisms; around 2015, there was a peak in cell therapy research represented by mesenchymal stem cells, while intra-articular injection drug delivery strategies and cartilage repair techniques gained widespread attention. Notably, after 2020, research related to extracellular vesicles rapidly emerged and formed close coupling with mesenchymal stem cell research, reflecting the transition from traditional cell therapy to cell-free therapeutic strategies. The knowledge map also reveals interactions among multiple research themes including synovial fluid microenvironment, cartilage defect repair, and angiogenesis, suggesting that osteoarthritis treatment research is moving towards a more comprehensive and precise direction. Particularly, as an emerging therapeutic carrier, extracellular vesicles display strong momentum in biogenesis mechanism research and delivery system development, which may indicate that cell-free therapy based on extracellular vesicles will play an important role in future osteoarthritis treatment. Author keywords typically represent the most representative terms chosen to explain research topics.

### Analysis of references

**Fig. 8:**
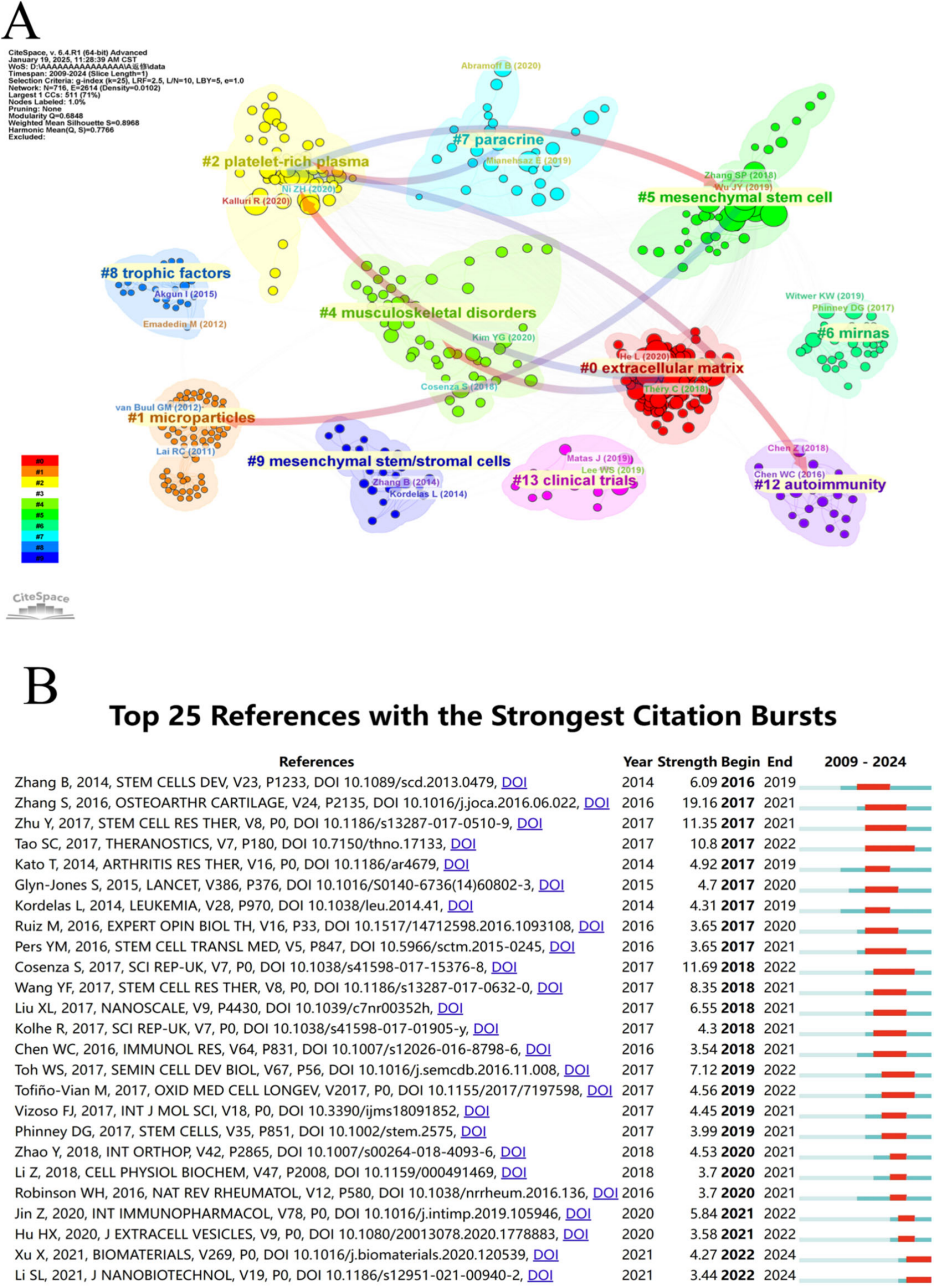
Reference analysis. **A** Co-cited reference clustering. References with similar themes or research directions are grouped into clusters, each assigned distinct colors. The establishment and development of certain clusters may depend on literature from other clusters as their theoretical foundation or methodological source. These dependency pathways are visualized through arrows in the diagram, illustrating how knowledge flows from foundational works to subsequent research. **B** Top 25 references with strongest citation burst. The blue bar represents the entire lifecycle of the keyword, while the red bar indicates the burst period of keyword research, detecting the start and end times as well as the burst intensity

Co-citation analysis of references is a crucial function of CiteSpace that enables the construction of knowledge maps to reflect the knowledge structure of research fields. In Fig. 8A, we present 11 major clusters in the field's reference co-citation analysis, with smaller clusters omitted. The colored arrows illustrate how newer research groups evolved from and built upon original research groups. An interesting research development chain: Early studies on mesenchymal stem cells (#5) and autoimmunity (#12) laid the foundation for the development of platelet-rich plasma (#2). This suggests that the development of platelet-rich plasma therapy drew from both stem cell therapy concepts and immunomodulatory mechanisms. Subsequently, research on platelet-rich plasma (#2) and musculoskeletal diseases (#4) jointly promoted the development of extracellular matrix (#0) research, reflecting researchers' growing recognition of the central role of the extracellular matrix in disease development and treatment. Meanwhile, platelet-rich plasma research also gave rise to research on paracrine (#7) mechanisms, indicating that scientists began to focus more on molecular mechanisms of treatment. This evolutionary pathway demonstrated the transition of osteoarthritis treatment research from cellular to cell-free therapeutic strategies, as well as the progression of therapeutic mechanism understanding from macroscopic to microscopic levels.

Citation bursts indicate high academic attention to certain studies during specific periods. As shown in Fig. 8B, we identified the most impactful 25 references characterized by dramatic increases in citations. Among these, the paper with the highest burst (burst strength = 19.16) value was authored by Zhang et al*.* [45]. Rats with bilateral femoral cartilage defects received weekly exosome treatment on one knee and PBS on the contralateral side. At weeks 6 and 12 post-surgery, exosome-treated defects manifested superior healing compared to PBS controls. This research offered the initial proof of the effectiveness of human embryonic MSCs-exos for cartilage repair and exhibited the usefulness of MSCs-exos as a readily available, 'cell-free' option instead of cell-based MSCs (mesenchymal stem cells) therapy. Furthermore, we observed that an article by Tao et al*.* proved that exosomes originated from miR-140-5p-overexpressing synovial mesenchymal stem cells could effectively treat OA, providing new therapeutic insights for OA [32]. This article initiated a surge in co-citations beginning in 2007 that persisted for 5 years. Remarkably, although the burst period for the majority of references has ended, there are still some references that are continuously experiencing citation bursts, suggesting continuous interest in these research topics in recent years. Additionally, citation frequency can reflect a paper's impact, with higher citation rates indicating greater academic significance in the area. Table 3 lists the leading 10 most-cited papers.

**Table 3:** The top 10 most cited works of literature on the search

Rank	Title	Journal	First Author	Publication year	Total citations
1	Exosomes derived from human embryonic mesenchymal stem cells promote osteochondral regeneration	Osteoarthritis and Cartilage	Zhang, S	2016	492
2	Mesenchymal stem cells derived exosomes and microparticles protect cartilage and bone from degradation in osteoarthritis	Scientific Reports	Cosenza, Stella	2017	446
3	MSC exosomes alleviate temporomandibular joint osteoarthritis by attenuating inflammation and restoring matrix homeostasis	Biomaterials	Zhang, Shipin	2019	376
4	miR-100-5p-abundant exosomes derived from infrapatellar fat pad MSCs protect articular cartilage and ameliorate gait abnormalities via inhibition of mTOR in osteoarthritis	Biomaterials	Wu, Jiangyi	2019	374
5	MSC exosome as a cell-free MSC therapy for cartilage regeneration: Implications for osteoarthritis treatment	Seminars in Cell & Developmental Biology	Toh, Wei Seong	2017	354
6	Comparison of exosomes secreted by induced pluripotent stem cell-derived mesenchymal stem cells and synovial membrane-derived mesenchymal stem cells for the treatment of osteoarthritis	Stem Cell Research & Therapy	Zhu, Yu	2017	308
7	Mesenchymal Stromal/stem Cell-derived Extracellular Vesicles Promote Human Cartilage Regeneration (*In Vitro*)	Theranostics	Vonk, Lucienne A	2018	267
8	Exosomes from embryonic mesenchymal stem cells alleviate osteoarthritis through balancing synthesis and degradation of cartilage extracellular matrix	Stem Cell Research & Therapy	Wang, Yafei	2017	244
9	Chondrocyte-Targeted MicroRNA Delivery by Engineered Exosomes toward a Cell-Free Osteoarthritis Therapy	Acs Applied Materials & Interfaces	Liang, Yujie	2020	239
10	Exosome-mediated delivery of kartogenin for chondrogenesis of synovial fluid-derived mesenchymal stem cells and cartilage regeneration	Biomaterials	Xu, Xiao	2021	230

## Discussion

### Basic information

Amidst the continual innovations in scientific scholarship, keeping pace with the latest research developments is more critical than ever. The proliferation of big data has led to an unprecedented volume of scholarly publications and data, resulting in growing difficulties for academics striving to monitor and assess recent scientific advancements. Nevertheless, by conducting statistical evaluations of scholarly information, bibliometric analysis offers a useful method for better understand research advancements, recognize growing areas of scientific interest, and forecast forthcoming scientific trajectories. From what we have gathered, this is the first bibliometric report on OA and EVs. Our analysis incorporated a sum of 366 scholarly articles, comprising 193 articles and 173 reviews. In terms of annual publication output, the period from 1991 to 2018 can be considered the first stage, characterized by a low number of publications, and no more than 10 papers were published in any given year. The years 2019 onwards mark the second phase, characterized by the steady worldwide emergence of top-tier studies in this domain. From 2022 onwards, the publication output has remained stable. The third stage began in 2022, when publication output stabilized and peaked in 2024. Among the main contributors in this area, China boasts the greatest quantity of scholarly output and total citations, closely followed by the United States, both being the principal drivers of research in this domain. Furthermore, it is noteworthy that notwithstanding the fewer publications, certain countries have an exceptionally elevated mean citations for each article. For instance, Singapore leads by a significant margin with an impressive 188.00, followed by Netherlands (165.00) and Ireland (76.00). The remarkably high average citation rate of Singapore can be ascribed to the issuance of various groundbreaking research reports by institutions such as the National University of Singapore and the Agency for Science, Technology and Research (A*STAR). For example, a study published in *Biomaterials* in 2018 found that exosomes extracted from MSCs improve cartilage repair through various mechanisms, including promoting proliferation, inhibiting apoptosis, and immunomodulation [33]. Toh et al. published a systematic review in *Seminars in Cell & Developmental Biology* in 2017, discussing the application prospects of MSCs-exos as cell-free therapeutics in cartilage regeneration and OA treatment [34]. In the same year, Wang et al. issued a scientific report in *Stem Cell Research & Therapy*, showing that exosomes originating from MSCs in embryos can mitigate OA by equalizing the production and breakdown of the extracellular matrix (ECM) of cartilage [46]. Additionally, Tan et al. published a systematic review in *Tissue Engineering Part B* in 2021 [47], summarizing the current status of MSCs-exos applications in animal studies of cartilage regeneration. From an institutional analysis perspective, Shanghai Jiao Tong University has published the highest number of papers. Despite this, it bears emphasizing that the extent of joint initiatives between academic establishments is comparatively minimal, with the majority of collaborations occurring at the country or territory level. In light of this, forging cooperative alliances among various academic organizations and investigative units is paramount for forthcoming studies. The periodical boasting the greatest quantity of printed articles is the *International Journal of Molecular Sciences*, while the journal with the highest number of citations is *Biomaterials*. De Girolamo, Laura from Aix-Marseille Universite and Ragni, Enrico from IRCCS Orthoped Inst Galeazzi have jointly published the highest number of papers, while Noel, Daniele from Universite de Montpellier had the highest citation rate.

### Current research status and hot spots

#### Mechanisms of action of cell-free therapy based on EVs for OA

Cell-free therapy based on EVs has surfaced as a groundbreaking approach in regenerative medicine, leveraging the curative capacity of cell-secreted bioactive substances, as opposed to the cellular components themselves, to promote tissue restoration and regeneration [48, 49], and offering an alternative to traditional cell-based therapies that require direct transplantation of living cells into the target tissue [28]. These vesicles, including exosomes and MVs [50], made up of various functional substances, such as proteins, lipids, and nucleic acids [20], which transport to affected cells within injured joint structures, subsequently altering their responses and capabilities [51, 52]. Among these alternatives, vesicles released by MSCs have display favorable medicinal impacts in multiple test scenarios, such as heart-related illnesses, sudden kidney failure, hepatic impairment, and lung tissue damage [52–56]. These vesicles influence immune system activity by decreasing the release of inflammation-related proteins, such as IL-1β and TNF-α, and by fostering an environment conducive to tissue regeneration [16]. Meanwhile, gene-modifying activities driven by miRNAs are essential for the medicinal properties of cell-free approaches, with EVs being enriched in miRNAs that can modulate the expression of genes involved in inflammation, autophagy, and other relevant pathways. Studies indicate these vesicles promote macrophage conversion from inflammatory M1 state to regenerative M2 form, supporting decreased joint inflammation [23, 57]. Additionally, studies reveal that EVs enhance cartilage matrix restoration in osteoarthritic tissue by reducing levels of proteins that break down matrix components, including MMP13 and ADAMTS5, thereby maintaining cartilage structure and delaying OA development [32, 46, 58, 59]. Moreover, EVs modify chondrocyte metabolic processes by boosting cellular energy production and ATP generation, thus strengthening their performance and stress resistance [60]. The cell homing properties of certain EVs, mediated by chemokines and other signaling molecules, allow for the recruitment of endogenous stem cells or progenitor cells to the site of injury, where they can participate in the tissue repair and regeneration process [61]. Some studies have also revealed that microparticles can upregulate the expression of type II collagen, suppress the expression of inflammatory markers and macrophage activation, protect chondrocytes from apoptosis, and improve cartilage parameters [30]. These microparticles exhibit therapeutic effects similar to those of exosomes, thus playing a positive role in the treatment of OA [62]. Beyond the direct actions of EVs, this treatment also enhances tissue regeneration through paracrine effects, with therapeutic cells secreting various forms of cytokines, growth factors, and other bioactive molecules that modulate the microenvironment of the damaged joint tissues, suppressing inflammation, promoting blood vessel formation, and modulating immune responses [63, 64]. Cell-free approaches target various OA mechanisms, offering an integrated solution for joint disease while advancing innovative regenerative treatments in orthopedic medicine [65, 66].

#### EVs in the treatment of osteoarthritis

OA, a common joint disorder, causes progressive degeneration that significantly diminishes patient well-being and daily functioning. As researchers seek to better understand the pathogenesis of this debilitating condition and develop more effective treatments, they have turned their attention to the role of EVs in the disease process. Exosomes and MVs, subclasses of EVs, have garnered significant attention in the pathogenesis and treatment of OA. The mechanisms underlying their roles have emerged as a focal point of research in this field. Cosenza et al. discovered that EVs derived from MSCs possess a chondroprotective effect, capable of mitigating IL-1β-induced chondrocyte apoptosis and matrix degradation [30]. Tao et al. identified key miRNAs, including miR-140-5p, EVs isolated from synovial fluid MSCs of individuals with OA. They found that these miRNAs promote chondrocyte proliferation and migration, suppress the expression of MMP-13 and ADAMTS-5, and are essential for EV-facilitated cartilage restoration [32]. Wu et al. further elucidated that MSC-derived EVs transport miR-100-5p and other miRNAs to inhibit the mTOR pathway in macrophages, thereby attenuating synovial inflammation and exerting an immunomodulatory mechanism [23]. Liu et al. further revealed that the lncRNA KLF3-AS1 in MSCs-exos could adsorb miR-206 by sponging, thus alleviating the suppressive impact of miR-206 on GIT1. Therefore, EVs mediate the restraining influence of the lncRNA-KLF3-AS1/miR-206/GIT1 axis [67]. Taken together, EVs, as essential intercellular communication mediators, regulate the functions of chondrocytes and immunomodulatory cells through the transfer of functional molecules such as non-coding RNAs. They are essential to the development and treatment of OA, emerging as a frontier and hotspot in precision medicine and regenerative medicine research.

### Future directions

#### Exosome engineering

Exosomes, a type of vesicles secreted by cells, have developed into a novel drug delivery vehicle owing to their distinctive advantages in various aspects, such as biocompatibility, stability, and ability to cross biological barriers [68, 69]. Exosomes possess a multitude of unique properties, for example capability to cross biological boundaries, strong blood circulation persistence, and precise targeting ability, rendering them outstanding drug delivery vehicles. These distinctive features can be attributed to their biological composition and tiny dimensions, typically ranging from 40 to 100 nm [68, 70]. Engineered exosomes, which have undergone surface modifications including genetic engineering and chemical alterations, demonstrate improved targeting abilities. These adjustments elevate regional concentrations, reduced immunogenicity, minimal toxicity, and strong engineerability, thereby enhance their potential for treating numerous medical conditions [71]. Beyond serving as drug delivery vehicles, exosomes have natural properties that support osteoarthritic cartilage restoration. These inherent capabilities include the mitigation of local inflammation, the promotion of anabolic metabolism, and the suppression of catabolic processes [27, 72]. Several genes within chondrocytes have been identified as closely related to the progression mechanism of OA. Consequently, genome editing is considered a possible therapeutic approach that could offer long-term benefits for patients suffering from this debilitating condition [73, 74]. Liang et al*.* genetically engineered the exosomal surface protein Lamp2b to express a chondrocyte-affinity peptide on the exosome exterior, allowing vesicles to locate cartilage cells and be retained within the joint after intra-articular injection. Moreover, he combination of exosomes and lipid vesicles allowed the resulting hybrid exosomes to encapsulate CRISPR/Cas9 plasmids meanwhile maintaining the chondrocyte-homing properties, thereby delaying cartilage degradation* in vivo* and accelerating bone tissue repair [75].

Chen et al. researched the relationship between FGF18 silencing and the short of assorted cartilage-specific genes with* in vivo* and* in vitro* studies of OA progression. They s effectively created a innovative* in vivo* approach founded on CRISPR/Cas9 technology to activate the FGF18 gene in OA chondrocytes on a genetic basis. This method endowed chondrocytes with targeting capabilities, thereby enhancing* in vivo* binding efficiency and biosafety [76].

Zhao et al. utilized engineered exosomes derived from subcutaneous (SC) adipose tissue-derived MSCs-exos to achieve targeted transport of miR-199a-3p into chondrocytes. This approach was feasible due to the relative ease of obtaining healthy donor subcutaneous adipose tissue. In an OA mouse model, this targeted delivery system displayed considerable cartilage regeneration [77].

Wan et al. designed a novel engineered exosome-based drug delivery system to alleviate OA symptoms. They chemically modified exosomes by acylating methacrylic anhydride with the amino groups at the exosomal membrane, forming olefin double bond-modified exosomes. Through acylation with the peptides' amino groups, targeting peptides were conjugated to the modified exosomes, resulting in peptide-exosome binding. The engineered exosomes, modified with the cartilage-affinity peptide WYRGRL, were encapsulated in a new light-crosslinked spherical gelatin methacryloyl hydrogel (GelMA) for OA treatment. The performance of the engineered exosomes abundant in the small inhibitor LRRK2-IN-1 (W-Exo-L@GelMA) was surveyed both in cell cultures and animal models. W-Exo@GelMA evidenced effective chondrocyte targeting and markedly suppressed the expression of genes related to inflammation and immunity in OA [78]. These innovative, in-depth and extensive studies have laid an extremely solid foundation for the cell-free treatment of OA.

Looking ahead, several challenges remain. To advance exosome-centered OA therapy, standardization of exosome isolation and characterization protocols must be achieved, effective regulatory processes must be implemented, and therapeutic efficacy must be validated through clinical trials [79].

#### New drug delivery system for EVs

Recently, the development of novel delivery systems has become a focal point of investigation in OA therapy. The treatment approaches for OA can be divided into two primary groups: pharmacological and non-pharmacological interventions. Pharmacological therapies can be further subdivided based on the route of administration, which includes oral, injectable, and topical modalities [80]. An ideal drug delivery platform should efficiently cross biological barriers and precisely release the necessary quantity of active compounds at the designated time and location. Utilizing naturally derived materials with excellent biocompatibility for targeted drug delivery to specific cells or molecules ensures the safety of the drug delivery vehicle [81–83].

The microenvironment of osteoarthritic joints is characterized by distinct pathological changes. These changes are relevant to factors such as pH levels, reactive oxygen species (ROS), and enzyme concentrations. Consequently, endogenous stimuli-responsive mechanisms that are sensitive to these conditions are currently under development. Nanoplatform-based drug delivery systems present innovative solutions for precise drug administration. These platforms enable enhanced drug accumulation and release kinetics at targeted disease locations, with the ability to respond to both endogenous and exogenous triggers for controlled therapeutic delivery. This strategy improves therapeutic uptake while reducing adverse effects throughout the body [81, 84]. To date, multiple delivery vehicles engineered for cartilage specificity have been created that facilitate the penetration of the compact ECM and improve therapeutic transport. Notably, nanoparticles incorporating chondrocyte-affinity peptides exhibit prolonged retention within the cartilage, causing them to become viable alternatives for the treatment of OA. In this burgeoning area of drug delivery study, the surface of exosomes is altered to boost their capabilities, facilitating site-specific drug delivery, along with* in vivo* visualization and tracing. Notably, nanoparticles incorporating chondrocyte-affinity peptides exhibit prolonged retention within the cartilage, making them promising candidates for the treatment of OA. Surface modification of exosomes in this innovative area of drug delivery research aims to augment their capabilities, facilitating targeted drug delivery,* in vivo* imaging, and monitoring [85].

Some researchers have attempted to establish an effective delivery strategy to enable exosomes to achieve continuous release in the superficial defect area [86]. According to a study carried out by Zhang et al*.*, an injectable hydrogel with robust bonding properties to the moist chondral tissue was prepared through the enzymatic cross-linking of alginate-dopamine, chondroitin sulfate, and regenerated silk fibroin (AD/CS/RSF). The interfacial shear strength of the hydrogel reached 120 kPa. By encapsulating exosomes within the hydrogel, endogenous BMSCs in a rat model were recruited to the lesion location through chemoattractant transduction cascades and induced to differentiate into chondrocytes, assisting in the restoration of chondral lesions in the trochlear groove. This research has yielded an encouraging bioengineered substance for individuals with shallow chondral lesions, potentially deliverable via minimally invasive arthroscopic techniques [87]. Chen et al. designed a bioscaffold for the delivery of MSCs-exos and fabricated a 3D-printed scaffold composed of chondrocyte ECM, gelatin methacrylate (GelMA), and exosomes with radial channels utilizing desktop stereolithography technology. In a rabbit osteochondral lesion model, the ECM/GelMA/exosome scaffold productively rehabilitated chondrocyte mitochondrial dysfunction, increased chondrocyte motility, as well as polarized the response of synovial macrophages towards the M2 phenotype. The 3D printed scaffold remarkably promoted cartilage restoration in the* in vivo* study. The approach may serve as an encouraging approach for the therapy of OA [88]. Zeng et al. designed a multifunctional hydrogel system inspired by mussels, which exhibited thermosensitivity, self-healing properties, and adhesive characteristics. This system enabled the co-delivery and complementary actions of MSCs-exos and icariin (ICA). The intra-articular injection system combining ICA and MSCs-exos is expected to be retained within the joint cavity, alleviate cartilage atrophy, and ensure proper cartilage thickness [27].

Future research will focus on developing novel EVs-biomaterial composite carriers, optimizing 3D printing processes, and improving their loading capacity and bioavailability. Furthermore, EVs modification strategies, such as lipid insertion metabolic labeling, and peptide CAQK-modified, are important means to enhance the pharmacokinetic properties of EVs [89–92]. Continued efforts to strengthen the fundamental research and industrialization exploration of exosome engineering, along with the establishment of standardized technical guidelines and translational pathways, will undoubtedly promote the widespread application of engineered exosomes in the precise diagnosis and treatment of OA. With the progress in nanotechnology, tissue engineering, and other fields, novel EVs delivery systems are anticipated to assume a more significant function in the identification and management of degenerative joint disease.

## Conclusion

In conclusion, this article provided a thorough examination of the research outlook and trends in the domain of cell-free therapy based on EVs in OA. This bibliometric analysis also uncovers the key research themes and hotspots, and prominent the importance of interdisciplinary collaboration, spanning areas such as biomaterials, tissue engineering, and nanotechnology, in driving innovation and translational advances in cell-free for OA.

However, the study also identifies potential gaps and challenges, such as the optimization of EVs engineering techniques and the evaluation of long-term safety and efficacy in clinical settings. Future studies need to concentrate on dealing with these limitations and exploring novel strategies for harnessing the therapeutic potential of EVs in OA management.

In summary, this quantitative literature assessment offers an informative asset for investigators, healthcare professionals, and decision-makers by presenting an evidence-based outlook on the present landscape and forthcoming trajectories of cell-free therapy based on EVs in OA. The findings emphasize the importance of fostering international collaboration, investing in interdisciplinary research, and translating basic science discoveries into clinical applications. As the realm persists in evolving, it is expected that cell-free therapy based on EVs will exert an increasingly crucial role in the development of innovative, personalized, and efficacious therapies for OA, eventually enhance patient results and life quality.

## Data Availability

The data underpinning this investigation was exclusively derived from the Web of Science (WoS) databases, and the original contributions of this study are presented within the article and its supplementary materials. Should there be a need for further inquiries, direct contact with the authors is encouraged.
